# Preliminary Attainability Assessment of Real-World Data for Answering Major Clinical Research Questions in Breast Cancer Brain Metastasis: Framework Development and Validation Study

**DOI:** 10.2196/43359

**Published:** 2023-03-23

**Authors:** Min Jeong Kim, Hyo Jung Kim, Danbee Kang, Hee Kyung Ahn, Soo-Yong Shin, Seri Park, Juhee Cho, Yeon Hee Park

**Affiliations:** 1 Department of Digital Health Samsung Advanced Institute for Health Science & Technology Sungkyunkwan University Seoul Republic of Korea; 2 Center for Research Resource Standardization, Research Institution for Future Medicine Samsung Medical Center Sungkyunkwan University School of Medicine Seoul Republic of Korea; 3 Center for Clinical Epidemiology Samsung Medical Center Sungkyunkwan University School of Medicine Seoul Republic of Korea; 4 Department of Clinical Research Design and Evaluation Samsung Advanced Institute for Health Science & Technology Sungkyunkwan University Seoul Republic of Korea; 5 Division of Medical Oncology Department of Internal Medicine Gachon University Gil Medical Center Incheon Republic of Korea; 6 Department of Intelligent Precision Healthcare Convergence Sungkyunkwan University Suwon Republic of Korea; 7 Department of Health Sciences and Technology Samsung Advanced Institute for Health Sciences and Technology Sungkyunkwan University Seoul Republic of Korea; 8 Department of Epidemiology and Medicine The Welch Center for Prevention, Epidemiology, and Clinical Research Johns Hopkins Bloomberg School of Public Health Baltimore, MD United States; 9 Division of Hematology-Oncology Department of Medicine, Samsung Medical Center Sungkyunkwan University School of Medicine Seoul Republic of Korea

**Keywords:** real-world data, preliminary attainability assessment, observational study, clinical data warehouse, PAR framework, brain metastasis, breast cancer

## Abstract

**Background:**

In recent decades, real-world evidence (RWE) in oncology has rapidly gained traction for its potential to answer clinical questions that cannot be directly addressed by randomized clinical trials. Integrating real-world data (RWD) into clinical research promises to contribute to more sustainable research designs, including extension, augmentation, enrichment, and pragmatic designs. Nevertheless, clinical research using RWD is still limited because of concerns regarding the shortage of best practices for extracting, harmonizing, and analyzing RWD. In particular, pragmatic screening methods to determine whether the content of a data source is sufficient to answer the research questions before conducting the research with RWD have not yet been established.

**Objective:**

We examined the PAR (Preliminary Attainability Assessment of Real-World Data) framework and assessed its utility in breast cancer brain metastasis (BCBM), which has an unmet medical need for data attainability screening at the preliminary step of observational studies that use RWD.

**Methods:**

The PAR framework was proposed to assess data attainability from a particular data source during the early research process. The PAR framework has four sequential stages, starting with clinical question clarification: (1) operational definition of variables, (2) data matching (structural/semantic), (3) data screening and extraction, and (4) data attainability diagramming. We identified 5 clinical questions to be used for PAR framework evaluation through interviews and validated them with a survey of breast cancer experts. We used the Samsung Medical Center Breast Cancer Registry, a hospital-based real-time registry implemented in March 2021, leveraging the institution’s anonymized and deidentified clinical data warehouse platform. The number of breast cancer patients in the registry was 45,129; it covered the period from June 1995 to December 2021. The registry consists of 24 base data marts that represent disease-specific breast cancer characteristics and care pathways. The outcomes included screening results of the clinical questions via the PAR framework and a procedural diagram of data attainability for each research question.

**Results:**

Data attainability was tested for study feasibility according to the PAR framework with 5 clinical questions for BCBM. We obtained data sets that were sufficient to conduct studies with 4 of 5 clinical questions. The research questions stratified into 3 types when we developed data fields for clearly defined research variables. In the first, only 1 question could be answered using direct data variables. In the second, the other 3 questions required surrogate definitions that combined data variables. In the third, the question turned out to be not feasible for conducting further analysis.

**Conclusions:**

The adoption of the PAR framework was associated with more efficient preliminary clinical research using RWD from BCBM. Furthermore, this framework helped accelerate RWE generation through clinical research by enhancing transparency and reproducibility and lowering the entry barrier for clinical researchers.

## Introduction

Brain metastasis (BM) is a major cause of mortality in patients with breast cancer, and it increases the difficulty of treatment. Advancements in treatment and the development of brain imaging technology have increased the survival of patients with metastatic breast cancer, leading to an increased incidence of BM [1-4]. Nonetheless, the opportunity to participate in prospective randomized clinical trials (RCTs) is typically only available to a limited number of patients with breast cancer brain metastasis (BCBM). Design challenges, including heterogeneity of patients, varying definitions of clinical end points, and different methods to assess these end points, have led to excluding most patients with BCBM from RCTs [5,6]. Consequently, while the incidence and survival duration have expanded, clinical research methods for BCBM remain limited [7].

Meanwhile, real-world evidence (RWE) in oncology has rapidly gained traction in recent decades, with the potential to answer clinical questions that cannot be directly or completely addressed by RCTs [8,9]. Integrating real-world data (RWD) into clinical research promises to contribute to more sustainable research designs, including extension, augmentation, enrichment, and pragmatic design [10]. To support the need for RWE generation, medical institutions have provided RWD through the construction of clinical data warehouses (CDWs) based on electronic health records (EHRs) [11,12]. Nevertheless, clinical research using RWD is still limited because of concerns regarding confidence in nonrandomized RWD analyses and the shortage of best practices for extracting, harmonizing, and analyzing RWD—practices that improve transparency and reproducibility [13,14]. To address these concerns, researchers have emphasized the importance of comprehensively understanding data representation and data content while clarifying research questions [15].

However, pragmatic screening methods to determine whether the content of a data source is sufficient to answer the research questions before conducting research with RWD have not yet been established. Accurate but oversimplified instructions could lead to confusion among clinical researchers who seek to select the optimal RWD source for their hypothesis and research purpose [16-19]. In detail, specifying the research question and determining the appropriate level for understanding massive and complex RWD is still challenging for clinical researchers. The vagueness inherent in this complex process at an early stage of research is one cause of concern and controversy regarding the utility of RWD for generating scientific evidence [13,17]. Therefore, effective screening methods are needed to determine whether the contents of a data source are sufficient to answer the research questions before conducting studies using RWD [20,21].

This study suggests a method to screen specific data sources for their ability to address a clinical research question at the preliminary step of research design and evaluate the method’s utility in assessing data attainability for BCBM.

## Methods

### Overview

In line with major clinical research questions on BCBM, this study evaluated screening performance using the Preliminary Attainability Assessment of Real-World Data (PAR) framework, which can assist clinicians in assessing data attainability from an RWD source at a preliminary stage of the study design. This study was divided into 2 phases. In the preparation phase, we first identified clinical questions related to current unmet needs according to the perspectives of clinical experts on BCBM for evaluation of the framework. In the evaluation phase, the working group assessed the preliminary data attainability of the listed clinical questions with a specific data source, using the PAR framework presented in the following section.

Data attainability was defined in this study as the availability of data for reconstruction and extraction required from a particular data source to conduct clinical research regarding sample size, data fields, and content.

### Data Source and Population

The Samsung Medical Center Breast Cancer Registry (SMC BCR) is a hospital-based real-time registry implemented in March 2021 that leverages the institution’s anonymized and deidentified CDW platform: the Data Analytics and Research Window for Integrated Knowledge (DARWIN)–C. The inclusion criteria for the SMC BCR comprise the intersection of three patient conditions: (1) visited at least one of the medical oncology, breast surgery, or radiation oncology departments, (2) diagnosed with breast cancer under codes C50 or D05 of the International Classification of Diseases codes, and (3) aged older than 15 years at enrollment [19]. The number of breast cancer patients in SMC BCR was 45,129, and the registry covered the period from June 1995 to December 2021. The SMC BCR consists of 24 base data marts that represent disease-specific breast cancer characteristics and care pathways. The registry provides clinical variables in the form of structured data, including demographics, diagnostic history, treatment information (operation, chemotherapy, and radiation therapy), laboratory test results, and featured data fields extracted from free-text records, such as pathology, radiology, or genomic laboratory reports [19].

For BCBM patient identification, we used a BCBM data mart in the SMC BCR. The BCBM data mart contains predefined and preprocessed data from patients (n=1443 up to December 2021) with at least one BM indicator after a breast cancer diagnosis. The indicators, defined by clinical experts, are as follows: craniotomy record (payment-based), whole-brain radiotherapy treatment (regions for radiation treatment are brain, whole brain, and partial), gamma knife (code for gamma knife), and intrathecal methotrexate treatment. The data quality of the BCBM data mart was evaluated using manual chart reviews performed by 2 clinical nurses with expertise in data management; data validity was greater than 98% via 10% random sampling.

### Participants

#### Clinicians Interviewed to Develop the Key Clinical Questions

Seven breast cancer experts from 6 hospitals in the Republic of Korea participated in a survey. Two breast cancer experts were interviewed in advance to define clinical questions regarding BCBM.

#### Working Group for Data Attainability Assessment

A working group was formed for data attainability testing. It comprised people with clinical expertise and at least 10 years of experience in clinical research across various interdisciplinary areas, including medical informatics and epidemiology.

#### Clinical Question Preparation and Validation

We established clinical questions through 2 rounds of interviews with 2 breast cancer clinical experts in April and June 2021 (Textbox 1). From the interviews, we identified clinical questions that reflected the most recent and internationally relevant unmet clinical needs in BCBM [21-26]. We conducted a survey of experts’ opinions to validate the clinical significance and the feasibility of these clinical questions. Seven breast cancer clinical experts participated in the survey from September 27 to October 5, 2021. In this survey, clinical experts scored clinical significance, research availability, data attainability, and method suitability using EHR data on a 5-point Likert scale (range 1-5). Multimedia Appendices 1 and 2 show the average scores and correlation analysis results. All data analyses and calculations were performed using Microsoft Excel (2016 version; Microsoft Corp). Graphs and plots were designed using R (version 4.0.2; R Foundation for Statistical Computing).

**List of clinical questions developed from interviews with experts.**

**Clinical question A**

Is there a difference in survival outcomes between the brain as the primary metastasis site and brain metastasis accompanied by systemic metastasis?

**Clinical question B**

Does systemic treatment for brain metastasis patients affect survival outcomes according to subtype?

**Clinical question C**

Does the timing of systemic treatment initiation affect survival outcomes when brain metastasis is accompanied by systemic metastasis?

**Clinical question D**

Is there a difference in the prediction of brain metastasis in patients who previously received trastuzumab alone for neoadjuvant therapy and in patients who received pertuzumab and trastuzumab for neoadjuvant therapy?

**Clinical question E**

Can any record of neurological symptoms described by patients be translated as a surrogate factor of brain metastasis?

#### PAR Framework

**Figure 1:**
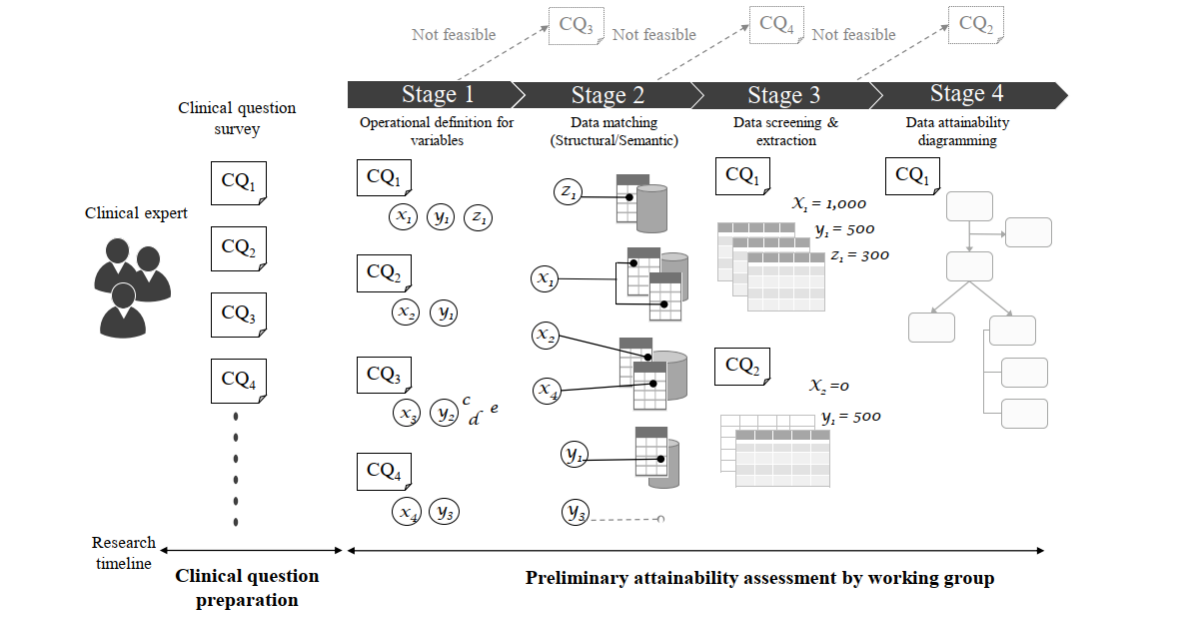
Illustration of the Preliminary Attainability Assessment of Real-World Data (PAR) framework and study design. CQ: clinical question.

The PAR framework was proposed to assess the data attainability of a data source from the perspective of a particular clinical question in the early research stages. The PAR framework has 4 sequential stages, starting with clarification of the clinical question (Figure 1).

#### Stage 1: Operational Definitions of Variables

In this step, we identify variables inherent in the clinical question, describe them in natural language, and clarify operational definitions of the variables at 2 distinct levels. One level is the research-variable level and includes dependent or independent variables at the description level of the research hypothesis. The other level is a data-variable level that can be used as an atomic condition to declare the research variable. For example, “overall survival (time)” can be one of the research variables that the researcher intends to observe, and “first diagnosis date” and “death date” are the data variables that constitute the research variable.

#### Stage 2: Data Matching (Structural/Semantic)

This stage involves matching the data variables with specific data fields in the data source selected as the research material. Data fields refer to stored data elements, such as the column name of the data table, that depend on the data source. By contrast, the data variable is a unified conceptual term. A one-to-one direct match has priority; however, a surrogate definition for the data variable is explored by combining the values from multiple data fields.

#### Stage 3: Data Screening and Extraction

Here, the actual data values are extracted and screened from the data source for each data variable identified in the previous stages.

#### Stage 4: Data Attainability Diagramming

Finally, we draw a diagram showing the extracted sample size according to the respective clinical question and matching process from the sub–data sets acquired in stage 3.

### Ethical Considerations

The study protocol was approved by the Institutional Review Board of the SMC (2021-09-036), which waived the need for informed consent as our study data were deidentified, and anonymized data were extracted from the CDW. This study followed the Standards for Quality Improvement Reporting Excellence (SQUIRE) guidelines [20].

## Results

**Figure 2:**
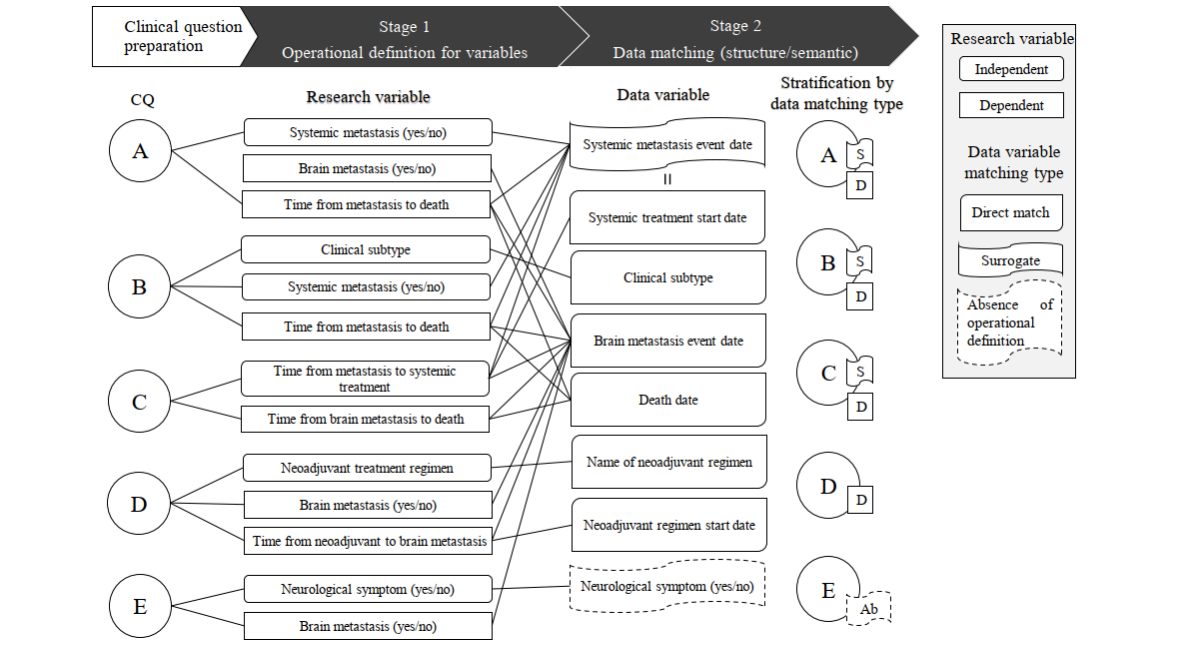
Results at stages 1 and 2 with the breast cancer brain metastasis clinical questions. CQ: clinical question.

The working group assessed data attainability pertaining to the 5 clinical questions through stages 1 to 4. In stage 1, the research and data variables were defined based on the SMC BCR data structure (Figure 2). For clinical question E, the variable “neurological symptoms” did not match with any structured or semistructured fields in the data source. Furthermore, terminological code systems covering “neurological symptoms” had not been applied in the EHR prior to the SMC BCR. Therefore, we concluded that clinical question E was not properly answered using our data source.

In stage 2, the data fields were matched with the defined operational data variables. At this stage, some questions required variable replacement to match data in the SMC BCR. The variables “BM event time,” “death date,” “neoadjuvant regimen start date,” and “neoadjuvant regimen name” were respectively coupled with corresponding data fields in the SMC BCR. By contrast, for “systematic metastasis event time,” a one-to-one direct match was not possible for any data field within the data source. Hence, we used “palliative treatment start date” as a surrogate variable, following clinical expert opinion, which reflected institutional treatment protocols.

In stage 3, the actual data values identified during the previous stages were extracted. The interdisciplinary team, including clinical experts, continued to validate the contents of the extracted data sets. Through this cross-validation, we confirmed whether the data were logically aligned with previously well-known clinical evidence. Data sets for 4 of the 5 clinical questions proposed by the breast cancer experts from a clinical perspective were extracted.

**Figure 3:**
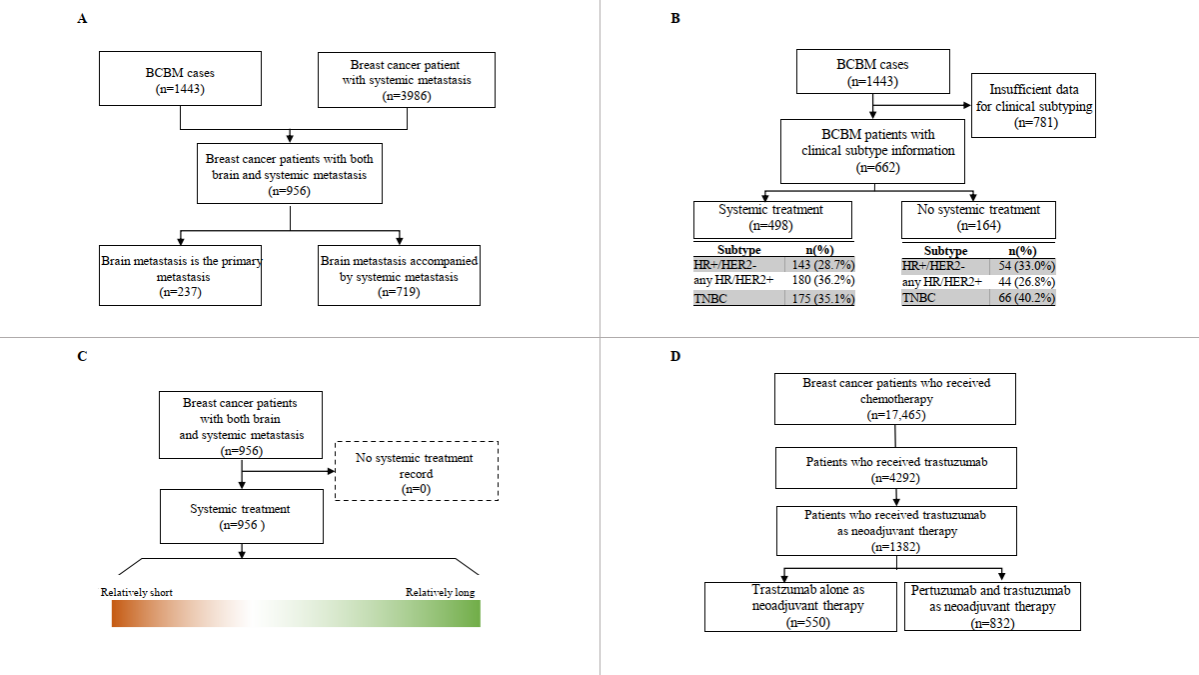
Data attainability for the clinical questions. Clinical questions were as follows. (A) Is there a difference in survival outcomes between the brain as the primary metastasis site and brain metastasis accompanied by systemic metastasis? (B) Does systemic treatment for brain metastasis patients affect survival outcomes according to subtype? (C) Does the timing of systemic treatment initiation affect survival outcomes when brain metastasis is accompanied by systemic metastasis? and (D) Is there a difference in the prediction of brain metastasis in patients who previously received trastuzumab alone for neoadjuvant therapy and in patients who received pertuzumab and trastuzumab for neoadjuvant therapy? BCBM: breast cancer brain metastasis; HER2: human epidermal growth factor receptor 2; HR: hormone receptor; TNBC: triple-negative breast cancer.

At the final stage, only clinical question D had a data set obtained from a directly matched data variable (Figure 3D). We gathered data for 4292 patients from 2006 to 2021 from the BCR for trastuzumab treatment. Among these patients, 1382 patients received trastuzumab treatment as a neoadjuvant treatment, 832 patients received combined trastuzumab and pertuzumab neoadjuvant treatment, and 550 patients received trastuzumab neoadjuvant treatment alone. The remaining clinical questions required substitutional variables owing to a lack of matched variables in the CDW system. Therefore, we obtained appropriate data sets for the other clinical questions from the data source with a defined surrogate variable.

To derive the data set for the clinical question “Does systemic treatment for brain metastasis patients affect survival outcomes according to subtype?” the BCBM mart and clinical subtyping information based on immunohistochemistry test results were used (Figure 3B). Clinical subtyping information was available for 662 patients who had test results for estrogen receptor, progesterone receptor, or human epidermal growth factor receptor 2 (HER2) items. HER2 results were curated based on supplementing the silver in situ hybridization or fluorescence in situ hybridization tests.

Systemic treatment records were only available for 498 patients. The number of patients with any hormone receptor (HR)/HER2 positive clinical subtype was 180 (36.2%), the number with triple-negative breast cancer (TNBC) was 175 (35.1%), and the number with an HR positive /HER2 negative clinical subtype was 143 (28.7%). In the subgroup with no systemic treatment records, 54 patients (33%) were classified into the HR positive/HER2 negative group, 44 patients (26.8%) into the HR/HER2 positive group, and 66 patients (40.2%) into the TNBC group.

In stage 2, the data-matching step, a surrogate data variable, “systemic treatment in a palliative setting,” was set to represent the presence of systemic metastasis. Among the 1443 BCBM patients, 956 patients were classified as having BM with systemic metastasis based on records of both BM-related and systemic treatment in a palliative setting. Of these 956 patients, 237 patients were classified as having BM that later progressed to systemic metastasis. Data attainability was verified for 2 clinical questions: “Is there a difference in survival outcomes between the brain as the primary metastasis site and brain metastasis accompanied by systemic metastasis?” and “Does the timing of systemic treatment initiation affect survival outcomes when brain metastasis is accompanied by systemic metastasis?” (Figure 3A and Figure 3C).

**Figure 4:**
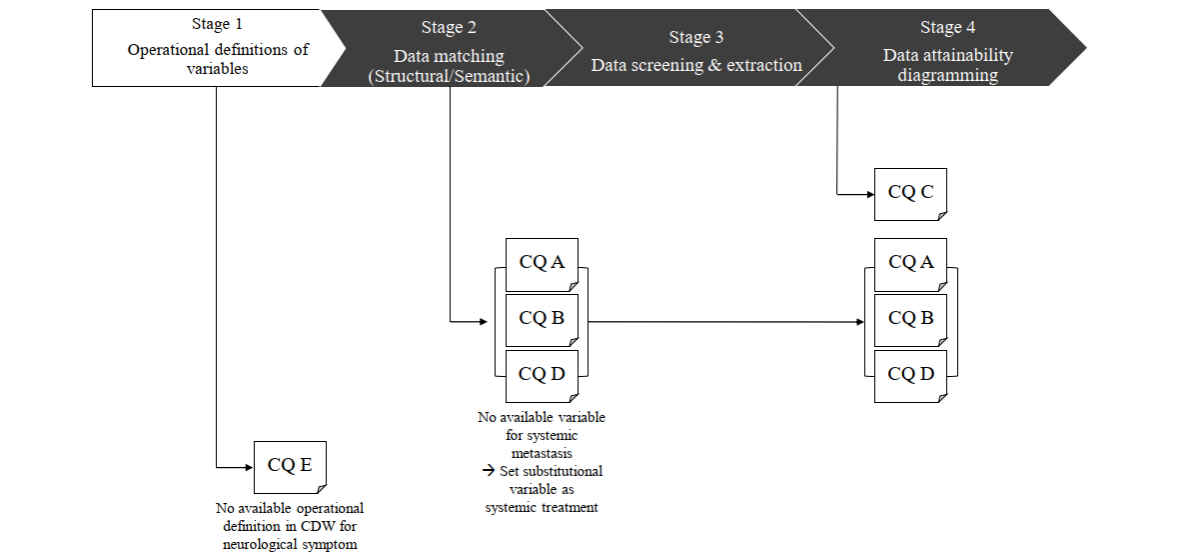
Preliminary Attainability Assessment of Real-World Data framework evaluation in stages 1 to 4. CQ: clinical question; CDW: clinical data warehouse.

During the process of clearly defining research variables in terms of data fields, the research questions stratified into 3 types. Among the 5 clinical questions suggested by clinical experts, we gathered data sets for 4 clinical questions using the stages suggested above. Only 1 clinical question could be answered using the matched direct data variables. However, the additional questions could be answered using surrogate variables (Figure 4).

## Discussion

### Principal Findings

In this study, we propose the PAR framework for data attainability screening at the preliminary step and evaluate its utility with clinical questions that reflect the most recent and internationally relevant unmet needs of clinicians in BCBM [21-26]. A survey was conducted to evaluate the clinical significance of the clinical questions. The mean score was 4.37 (range 3.57-5.00). We found that the correlation between scores given by experts was higher for the questions with higher average scores (Multimedia Appendices 1 and 2). RWE generation has received attention in the BCBM therapeutic area owing to limited clinical trial opportunities despite increasing clinical importance. However, incomplete gold standards for RWD study protocols and the unpredictable “hidden labor” of the secondary use of clinical data serve as barriers when clinical researchers attempt to design the most suitable methods to address their research questions [16,17]. We identified particular gaps when SMC built a site-specific CDW platform and developed a BCR as its first implementation case. After the release of the SMC BCR, clinical researchers attempted to generate RWE by using this registry, especially in areas with significant unmet needs, such as BCBM. Nevertheless, clinical research using RWD has not been as successful as expected in our experience despite the fact that well-known technical barriers are addressed; the CDW’s functional user interface provides clinicians with access to nonidentified and anonymous high-quality data sets. The greatest challenge faced by clinical researchers at the next step was securing a sufficient understanding of the data to avoid information distortion during the research data preparation stage. Therefore, we proposed the PAR framework to assess the feasibility of research at the preliminary stage and evaluated this framework with BCBM clinical questions with various entry points. To the best of our knowledge, a systematic framework to explore research feasibility at the conjunction between the data source and clinical questions has yet to be presented. Without such a framework, sample size, data fields, and content have not been properly accounted for. The methodology of this study can contribute to the acceleration of RWE generation by strengthening the transparency and reproducibility of the RWD research process and lowering the entry barrier for clinical researchers.

Clinical researchers with research questions derived from empirical insight in clinical practice have difficulties securing an understanding of accumulated RWD and solidifying their study designs. In contrast to conventional medical research methodologies, securing the reliability and validity of research variables for RWD studies is designed at a post– rather than pre–data collection stage. In addition, RWD is a conceptual collective term encompassing all data obtained through health care activities, and the content varies by data source [27]. Therefore, research using RWD requires significant and iterative effort prior to formal hypothesis testing, including selecting an appropriate data source, curating the data, repurposing it, and preprocessing it [28,29]. As a result, local information system expertise and deep content knowledge are often required to understand the idiosyncratic manner in which data sources are captured and stored [17,30]. Depending on the level of the data structure, data extraction frequently requires a high level of technical training as well. Through the process of matching research variables with data fields, the PAR framework illustrates how the process of clarifying research questions refines the range of data that should be analyzed before addressing a specific research question. Consequently, time and effort could be greatly reduced to ensure understanding of the selected data source.

The advantage of the PAR framework is that the results of stages 1 and 2 are explicitly communicated and can be reused. For example, in clinical question D, the research variable “time from neoadjuvant to BM” can be measured as the time between “neoadjuvant regiment start date” and “BM event date.” This level of operational definition can be reviewed and reused by peers with clinical expertise regardless of the storage structure of the data source. Above all, this reusability-enhancing reproducibility is further extended when applied to data sources that adopt not only institution-specific structures but also common data models, such as the Observational Medical Outcomes Partnership (OMOP) common data model [31] or the Informatics for Integrating Biology & the Bedside (i2b2) data model [32]. As specific data field conditions and query code levels with standardized data models and vocabularies can be reused, the accumulation of operational definitions of data levels can be considered an additional knowledge base. Furthermore, the accumulation of these definitions enhances consistency in the conduct of RWD research by enabling discussion regarding content validity in a more comparative manner. Accordingly, connecting formative efforts in the RWD research process, from data storage to processing, is a promising way to ensure reliability of research outcomes [33].

CDWs contain clinical data from EHRs for retrospective analysis to enable clinical researchers to utilize RWD for research directly, and the scope of RWD use has significantly expanded over the past few decades [31-34]. However, we identified several challenges while conducting RWD studies using the SMC BCR with clinical questions. A deficiency in the exact data variables for the questions recognized by clinical researchers was detected in stage 1. Researchers need to reassess stages 1 and 2 when no directly matching data field exists for a research variable. A surrogate definition could be considered as a substitution. For example, we adopted an indirect variable to represent systemic metastasis using the data for systemic treatment and used the start date of the palliative treatment cycle as the date of metastasis. Since most metastatic breast cancer patients with systemic metastasis receive systemic treatments, data on these indirect variables were readily available from the SMC BCR. However, when using an indirect variable as a surrogate, the validity of the research outcome is lower than when using a direct variable, which could be a primary limitation of RWD studies. Alternatively, if the variables will be frequently used for the generation of RWE, construction of featured data marts based on raw data and local practice rules is recommended. Since the SMC CDW has a well-constructed BCR with a BM mart, it was relatively easy for the investigators to extract the necessary data variables.

Additionally, it was not possible to extract key variables for clinical question E. This result aligned with the experts’ survey scores for the least feasible and least suitable questions using EHR data. Symptoms described by patients were only recorded in EHRs as free text, and there was no specific template or location. It was not possible to preprocess this information using a rule-based semantic engine for the CDW. Despite capturing symptoms of patients’ complaints, integrating the information into the CDW was difficult, because the location and template of the data were not aligned across departments. Moreover, the terminology for neurological symptoms is not standardized and is subject to a relatively high level of cultural dependency. To address this, integrating other types of data, such as prospective cohort studies or patient-generated data from mobile or watch-type devices, can be considered. If investigators continue to track patient-reported outcomes through cohort studies and integrate this information with other variables in CDWs, the use of RWE based on CDW data can be increased.

### Limitations

This study is limited in its generalizability. We assessed a single clinical domain, BCBM, and extracted data from a single data source, the SMC BCR. Further application of the PAR framework in different domains or with different types of RWD will be needed to develop the framework. However, the PAR framework and training case presented in this study could help guide clinical researchers in assessing preliminary attainability for future studies using RWD.

### Conclusions

We proposed and evaluated a PAR framework to assess data attainability to answer major clinical research questions in BCBM. The adoption of the PAR framework is associated with improved efficiency in clinical research using RWD in the preliminary stage. This framework could contribute to improving the quality of RWD-based clinical research by enhancing its transparency and reproducibility.

## Appendix

Multimedia Appendix 1 Comparison of expert assessments for each clinical question was conducted by surveying 7 experts' opinions from September 27th, 2021 to October 5th, 2021.

Multimedia Appendix 2 The statistical analysis of the survey results utilized correlation analysis between the mean value of each score and the SD.

## Abbreviations

BCBM: breast cancer brain metastasis
BCR: breast cancer registry
BM: brain metastasis
CDW: clinical data warehouse
DARWIN: Data Analytics and Research Window for Integrated Knowledge
EHR: electronic health records
HER2: human epidermal growth factor receptor 2
HR: hormone receptor
PAR: Preliminary Attainability Assessment of Real-world Data
RCT: randomized clinical trial
RWD: real-world data
RWE: real-world evidence
SMC BCR: Samsung Medical Center Breast Cancer Registry
TNBC: triple-negative breast cancer

## Data Availability

The data used for this study were extracted from a real-time breast cancer registry and breast cancer brain metastasis data mart integrated into the Data Analytics and Research Window for Integrated Knowledge–C clinical data warehouse of Samsung Medical Center. The data presented in this study are available on reasonable request from the corresponding author.
