# Supplementary figures and images for: Preliminary Attainability Assessment of Real-World Data for Answering Major Clinical Research Questions in Breast Cancer Brain Metastasis: Framework Development and Validation Study
Source: J Med Internet Res. 2023 Mar 23;25:e43359. doi: 10.2196/43359 (PMC10131620; doi:10.2196/43359)

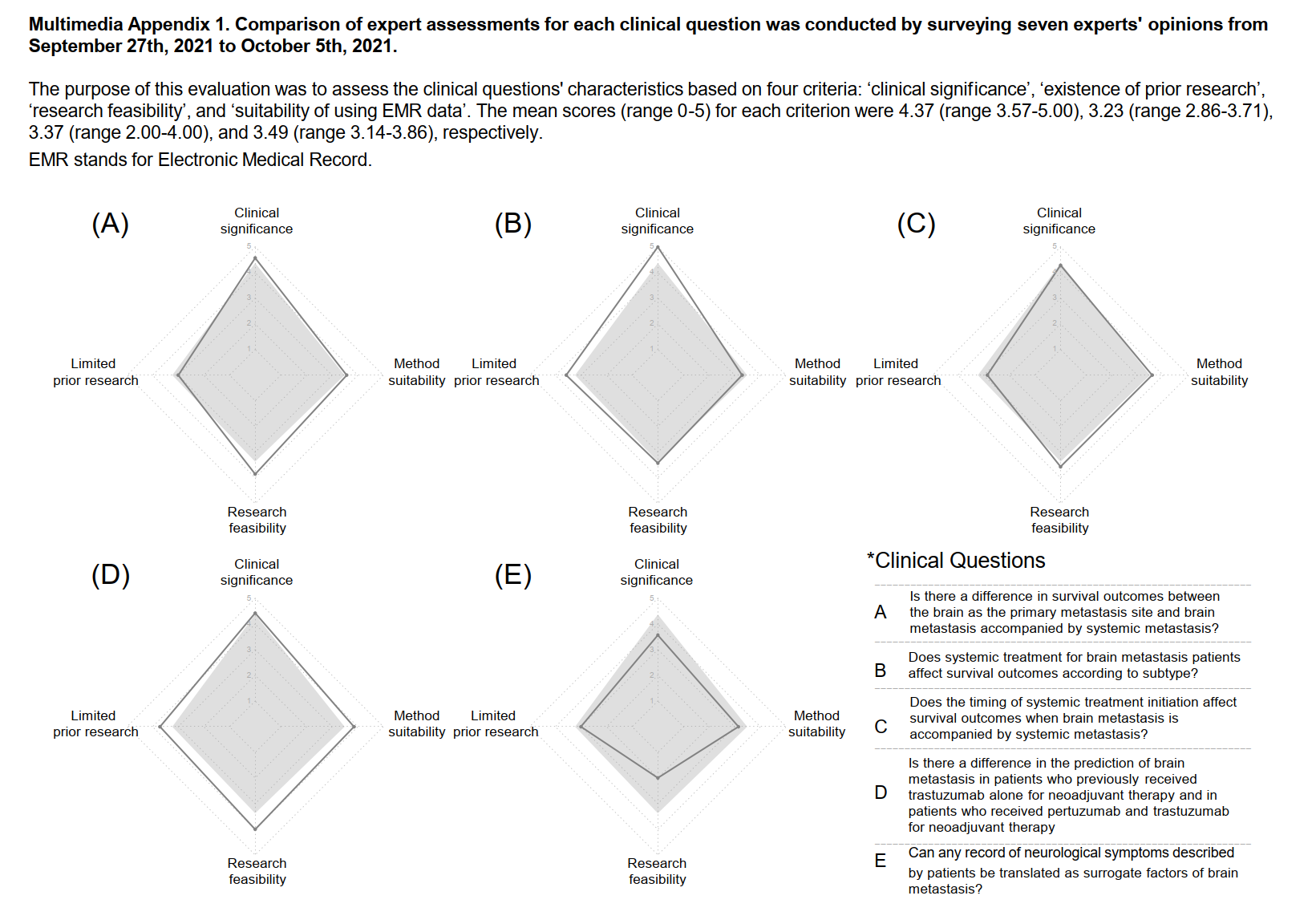

Supplement: Multimedia Appendix 1 [file jmir_v25i1e43359_app1.png]

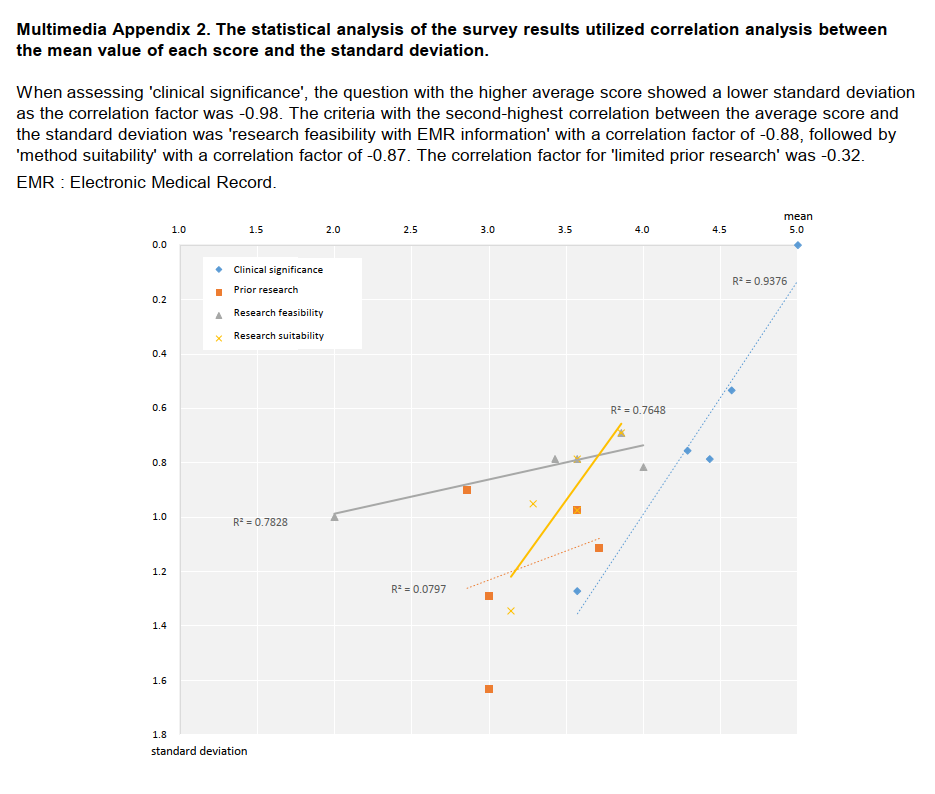

Supplement: Multimedia Appendix 2 [file jmir_v25i1e43359_app2.png]
